# An Overview of Data Routing Approaches for Wireless Sensor Networks

**DOI:** 10.3390/s120403964

**Published:** 2012-03-27

**Authors:** Mohammad Hossein Anisi, Abdul Hanan Abdullah, Shukor Abd Razak, Md. Asri Ngadi

**Affiliations:** Department of Computer Systems and Communications, Universiti Teknologi Malaysia (UTM), Johor 81310, Malaysia; E-Mails: hanan@utm.my (A.H.A.); shukorar@utm.my (S.A.R); dr.asri@utm.my (M.A.N.)

**Keywords:** Wireless Sensor Networks, data routing, energy, fault-tolerance, delay

## Abstract

Recent years have witnessed a growing interest in deploying large populations of microsensors that collaborate in a distributed manner to gather and process sensory data and deliver them to a sink node through wireless communications systems. Currently, there is a lot of interest in data routing for Wireless Sensor Networks (WSNs) due to their unique challenges compared to conventional routing in wired networks. In WSNs, each data routing approach follows a specific goal (goals) according to the application. Although the general goal of every data routing approach in WSNs is to extend the network lifetime and every approach should be aware of the energy level of the nodes, data routing approaches may focus on one (or some) specific goal(s) depending on the application. Thus, existing approaches can be categorized according to their routing goals. In this paper, the main goals of data routing approaches in sensor networks are described. Then, the best known and most recent data routing approaches in WSNs are classified and studied according to their specific goals.

## 1. Introduction

Current advances in micro-electromechanical systems (MEMS), low-power wireless communications, low-power analog and digital electronics that have led to the development of low-cost and low-power sensor nodes that are small in size is receiving increasing attention. Sensor nodes have the ability to sense the nearby environment, perform simple computations and communicate within a small region. Although their capacities are limited, combining these small sensors in large numbers provides a new technological platform, called Wireless Sensor Networks (WSNs). WSNs provide reliable operations in various application areas, including environmental monitoring, health monitoring, vehicle tracking system, military surveillance and earthquake observation [1].

They have been suggested for many applications where a large distribution of small and low-cost sensors is used in a vast area to sense the environment to detect events, gather information and transfer the information to a sink hop-by hop through the sensor nodes themselves. At each node both processing and the transmission of the information consume, more or less, some energy, which is usually supplied from the limited battery-based energy sources of the nodes themselves. Since the deployment of the sensors is usually done once for a long period of time, it is desired that the energy supply of the nodes last for a long time. Not only is the energy of individual nodes important during the lifetime of the network, but also the balance of the energy all across the network is equally important for full coverage of the area as well as finding a route along the path towards the sink.

Today, such networks have many applications in different scenarios such as battlefields, intelligent streets and highways, medicine, *etc.* [2–4]. Also, according to the nature of sensors, we can mention some advantages of sensor networks such as fast deployment in emergency situations, capability to be used in dangerous situations and low cost for information gathering over a long time. However, due to the limited sources of energy, delivering sensory data to the sink necessitates an efficient data routing solution. In *ad hoc* networks, routing protocols are anticipated to perform three main functions:

1. Determining and detecting network topology changes like node and link failures.
2. Maintaining network connectivity.
3. Computing and discovering appropriate routes.

To achieve these objectives, numerous solutions such as flooding, *ad hoc* clustering, geometric spanners, *etc* have been used [5–7]. However, in sensor networks, it is obvious that most of the *ad hoc* routing protocols are not suitable since the most important form of traffic in such networks is many to one and all the nodes normally report to a single Base Station (BS) or fusion center. Nevertheless, some qualities of these protocols can be related to the features of sensor networks, such as multi-hop communication and Quality of Service routing. Depending on the network structure, routing in WSNs can be classified into:

### Flat-based or Data Centric routing

Examples of this class are Sensor Protocols for Information via Negotiation (SPIN) [8], Minimum Cost Forwarding (MCF) [9], Directed Diffusion (DD) [10] or those mentioned in [11–12].

### Hierarchical-Based or Cluster-Based Routing

Energy efficient, weight-clustering algorithm (EWC) [13], TEEN (Threshold sensitive Energy Efficient sensor Network protocol) [14], Self-organizing Protocol (SOP) [15] or the systems described in [16–18] belong to this category.

### Location-Based Routing

Energy Aware Greedy Routing (EAGR) [19], Geographic and Energy Aware Routing (GEAR) [1], Geographical Adaptive Fidelity (GAF) [20] are some examples of this class of sensor networks routing protocols.

Data-centric protocols are query-based and work based on the naming of requested data. In this type of routing protocols, a query will be sent from a sink to certain regions in the network and it waits for the responses from the sensor nodes. Such queries have different attributes and data will be returned according to the specified attributes. In this area, the authors in [6] proposed a data-centric routing scheme based on flooding which uses only 1-hop neighbor information. In this scheme, to reduce the number of messages and conserve more energy, at each hop, just a subset of neighbor nodes is selected to rebroadcast the flooding message. The data-centric protocol in [21] makes use of historical link states and link quality estimation to route data toward the sink. It generates a connectivity graph according to the result of link quality estimation and considers dynamic windows for discovering historical link states. In [22] the authors proposed a topology aware routing (TAR) protocol which encodes a network topology to a low dimensional virtual coordinate area where hop distances between the nodes are maintained. According to accurate hop distance comparisons, TAR uses greedy forwarding to find the proper neighbor which is one hop closer to the sink and obtain high packet delivery ratio. In [7] a geometric spanner for static wireless *ad hoc* networks is proposed, which can be used in a localized manner. In this scheme, by utilization of a connected dominating set and the local Delaunay graph, an efficient network backbone is constructed and the communication cost is reduced.

Hierarchical algorithms separate the nodes into sub regions called clusters in order to segregate the areas of the monitoring environment. To allow communication between the clusters, a leader (cluster-head) is selected from each cluster. Leaders are then responsible for the management (data aggregation, queries dispatch) and transmission of the collected data within the region they control. Hierarchical or cluster-based routing usually consists of two layer routing: one layer relates to cluster head selection and the other one for routing. The main goal of these protocols is energy conservation by participation of the nodes in multi-hop communications [17]. Cluster Overlay Broadcast (COB) [23] works with route request messages and route reply messages flooded only by cluster heads. While receiving a route reply message, a cluster head labels itself as active for the related session. However, the route generated by this approach can only be used once. In [24] a clustering routing approach based on a Bayesian game is proposed. In this approach, to reduce the energy consumption, a static game of incomplete information in converted into a static game of complete information. In addition, both cluster head distribution and remaining energy of the nodes are taken into consideration in the design of the proposed routing algorithm. In [25] the authors proposed an intelligent system for controlling buildings and providing energy saving services. In this scheme, they used a self-clustering sensor network and proposed a node type indicator based routing (NTIR) protocol to support sensor network requirements and prolong the network lifetime.

Location-based algorithms relay on the use of a node's position information to find and forward data towards a destination in a specific network region. Position information is usually obtained from GPS (Global Positioning System) equipment or by exchanging some positional information. In recent years, several location-based routing approaches have been proposed. In [26] the authors proposed a 3D geographical routing (3DGR) scheme which uses the location information of the nodes to route data from source nodes to destinations by considering path quality and reliability. Moreover, the geographic routing algorithm which is proposed in [27] makes use of virtual position as the intermediate location of all direct neighbors of a node. In this scheme, instead of using nodes' real locations, the virtual positions are utilized in selecting the next hop. The so-called energy-aware interference-sensitive geographic routing (EIGR) protocol [28] aims to reduce the average energy consumption of networks and mitigate interference. For navigating data toward the sink, EIGR considers an anchor and selects the links with minimum interference from energy-efficient relay area for data transmission.

There are several surveys reviewing different aspects of WSNs such as energy-efficient design, MAC layer techniques, *etc.* [29,30]. Particularly, routing approaches in WSNs are surveyed by several papers, but none of them has categorized and described the existing routing approaches according to their goals. The authors in [31] explain and analyze the general routing strategies proposed for sensor networks. Likewise, routing protocols based on their structures are explained in [32] and [33]. The paper [34] describes secure routing protocols in WSNs. Also, energy-efficient routing protocols are classified and explained in [35] according to their energy-efficient mechanisms. Moreover, the authors in [36] provided an overview of existing fault-tolerant routing protocols in WSNs and classified the reviewed approaches into retransmission-based and replication-based protocols. To best of our knowledge, this paper is the first effort to classify and review the existing data routing approaches in WSNs according to their goals.

In this review article, we describe the main goals of data routing approaches in WSNs. Further, we classify the most famous and recent data routing approaches in WSNs according to their goals and attitudes and overview their mode of operation. In the next part, we discuss the three main goals of data routing in WSNs, including energy conservation, fast delivery and fault-tolerance.

## 2. Routing Goals

In WSNs, most of the proposed data routing approaches try to achieve a specific goal according to the application at hand. These goals can be categorized into three main categories: energy conservation, fast delivery and fault-tolerance. Although, the energy constraint nature of WSNs should be considered by every approach, most of the data routing approaches mainly focus on one of the mentioned three goals. In other words, they have been proposed for an application in which one of these goals is the main requirement. For example, the main objective of real-time routing protocols is to completely control the network delay; thus, they need fast data delivery. On the other hand, in some sensor applications, successful message delivery between source and destination is essential; hence, they require fault-tolerant data routing. Furthermore, some approaches considered a trade-off between these goals to satisfy required Quality of Service [37,38]. Therefore, we have classified the main goals of the existing data routing approaches in WSNs as follows:

### 2.1. Energy Conservation

The most complicated limitations in the design a WSN are those concerning the minimum energy consumption required to drive the circuits and possible micro-electromechanical devices. When miniaturizing the node, the energy of the power supply is the principal concern. Existing technology supplies batteries with about 1 J/mm^3^ of energy, while capacitors are able to attain as much as 1 mJ/mm^3^. If a node is considered to have a moderately short lifetime, for instance, a few months, a battery is a reasonable solution. However, for nodes which must be able to produce sensor readings for lengthy periods of time, a charging technique for the supply can be better. Presently, research groups are researching the use of solar cells to feed capacitors with photocurrent from the light. Solar flux can supply power density of about 1 mW/mm^2^. The energy efficiency of a solar cell ranges from 10 to 30 percent in contemporary technologies, offering 300 mW in full sunlight in the ideal case scenario for a 1-mm solar cell working at 1 V. Series-stacked solar cells should be used to provide proper voltages.

Sensors can operate with 1 nJ per sample, and recent processors are able to do computations using less than 1 nJ per command. For wireless communications, the main alternative technologies are based on RF and optical transmission methods which each have their own benefits and disadvantages. RF has a problem because the nodes may present very restricted space for antennas, thus requiring very short-wavelengths (for example, high-frequency) transmission, which must then tolerate very high attenuation. Consequently, communication in those system is not at this time compatible with low-power functions. Recent RF transmission mechanisms use approximately 100 nJ per bit for a transmission distance of 10 to 100 m, making communication very costly in comparison with data acquisition and processing [39–41].

In WSNs, where sensors are capable of sampling, processing and data transmission, the transactions among these tasks is an important issue in power handling. Sensor nodes can use up their limited supply of energy performing the mentioned parameters in a wireless environment. As such, energy-conserving forms of communication and computation are essential. Therefore, sensor node lifetime shows a strong dependence on battery lifetime. In a multi-hop WSN, each node plays a dual role as data sender and data router. The malfunctioning of some sensor nodes because of power failure can cause significant topological changes and might require rerouting packets and reorganizing the network. Thus, the key challenge in data routing is conserving the sensor energy, so as to maximize their lifetime, and much research on this topic is in progress [29,39,42–44].

### 2.2. Fast Delivery

Timely delivery of data is required in many sensor network applications such as real-time target tracking in battle environments and emergent event triggering in monitoring applications. In such applications, latency cannot be tolerated and data should be delivered within a certain period of time from the moment they are sensed; otherwise, the data will be useless. Also, bounded latency for data delivery is another condition for time-constrained applications [45].

In some real-time applications, data delivery is done based on a deadline by which data should be received at the BS. In this case, any message which received at the BS after the deadline is ignored. However, in some other real-time applications, there is no specific deadline for data delivery and a portion of data can be received at the BS after the deadline. In this situation, the part of data which is received after the deadline is considered the *miss ratio*, and some data routing approaches only focus on reducing the *miss ratio* by prioritizing the time-critical messages. Additionally, it should be noted that the principal goal of data routing approaches for these application is to control the network delay, but simultaneously they try to reduce energy consumption in transmission, in order to extend the network lifetime as much as possible under the delay constraints [11,46–48].

### 2.3. Fault-Tolerance

In WSNs, fault tolerance mechanisms should be considered in both nodes and links. As sensor nodes are battery enabled devices, they may lose their power and die quickly. However, the failure of some sensor nodes should not affect the overall task of the sensor network. If many nodes fail, MAC and routing protocols must accommodate formation of new links and routes to the data collection base stations. This may require actively adjusting transmitting powers and signaling rates on the existing links to reduce energy consumption, or rerouting packets through regions of the network where more energy is available. Additionally, such node failures may disrupt the connectivity of the network, therefore multiple levels of redundancy may be needed in a fault-tolerant sensor network. On the other hand, in a multi-hop sensor network, communicating nodes are linked by a wireless medium. Generally, the needed bandwidth of nodes' information will not be high, on the order of 1 to 100 kb/s. In such an area, the conventional problems associated with a wireless channel such as fading and high error rate have negative effects on data transmissions between sensor nodes and sink and may lead to packet loss. A packet loss has a direct effect on the accuracy of the results, therefore, a data routing approach in WSNs should be able to handle such faults, too [36,49,50].

## 3. Data Routing Approaches in WSNs

In this section, we study the data routing approaches in WSNs according to their main goals.

### 3.1. Energy-Efficient Approaches

In general, energy-efficiency is improved by utilization of two methods: first, *Energy balancing* in which overloading and multi-functioning of some specific nodes is prevented and the residual energy of the sensor nodes is monitored. Second, *Energy consumption reduction* to mitigate the average energy consumption of network by using different methods such as clustering, reducing the number of messages, reducing overheads and cooperative communications. In the following, we provide an overview of various approaches proposed in the literature under each category.

#### 3.1.1. Energy Balancing

In [51] the authors propose a mechanism for energy distribution in WSNs. They criticize the use of a stationary sink and multi-hop relaying as unbalancing energy expenditure. Hence, they have used multiple mobile sinks for solving the problem and proposed a scheme for correct positioning of the sinks and efficient data collection in the network. In general, the proposed approach address two problems: the ability to place a data collector in any region within the sensing environment, moving the data collector and placing it on tracks spanning the sensing area and discovering a search space for data collector locations.

In their proposed approach, the sinks can be replaced with any other position in the sensing area and they can be moved anywhere to track the events. Moreover, by utilization of linear programming, the proposed algorithm finds the discovery space of the sinks based on the remaining energy and future residual energy of the nodes. Furthermore, it finds the optimal locations of the data collectors and the flow paths and selects the one in which the total energy is minimum. In order to achieve this, the following function is proposed:

$$\alpha \;E_{\text{Min}}-\beta \;E_{\text{toal}}$$

Where, *E_Min_* is the minimum residual energy over all nodes at the end of each phase of the approach and *E_total_* is the whole energy consumption of each phase. Also, *α* is defined greater than *β* as below:

$$\alpha =1\;\text{and}\;\beta =\frac{1}{\sum_{0\leq i\leq N}E_{i}}$$

where *E_i_* is a constant; thus, 0 < *β E_total_* < 1 and any increase to *E_Min_* controls the reductions of *E_total_*. Therefore, in this approach, the energy level of the nodes is taken into consideration, the energy of the network is balanced and thus, the network lifetime is prolonged.

Geodesic Sensor Clustering (GESC) [52] is an energy-efficient distributed clustering approach which is able to balance energy among the nodes according to the network structure and residual energy of the nodes. Clustering and cluster head selection mechanisms in this approach are done hierarchically. A cluster member transmits its data to its cluster head and the cluster heads after performing data aggregation send their data to another cluster head.

Moreover, cluster head selection in GESC is carried out according to the significance of the nodes. It finds the significance of the nodes by performing some calculations. The node significance index *NI*(*υ*) in this approach is calculated as follows:

$$NI(\upsilon )=\sum_{u\neq \upsilon \neq w\varepsilon V}\frac{\delta_{uw}(\upsilon )}{\delta_{uw}}$$

In the equation, *δ_uw_*(*υ*) is the number of minimum hop paths from *u* to *w* wherein some vertices *υ ε V* are participated. Also, *δ_uw_* denotes the number of minimum hop routes from *u* to *w*.

The significance of a node depends on its ability to reach other nodes through the shortest paths. In GESC, each node discovers its one hop and two hop neighbors along with their residual energy by broadcasting an initialization packet. Then, for selecting its cluster head candidates, it seeks the list of its neighbors and selects the one hop neighbors which can cover at least one two hop neighbor. After detecting the cluster heads candidates, among the set of selected cluster heads, it selects the node with highest residual energy as the final cluster head. This cluster head selection is updated according to the network topology. With this strategy, GESC is able to distribute the energy consistently.

**Figure 1.**
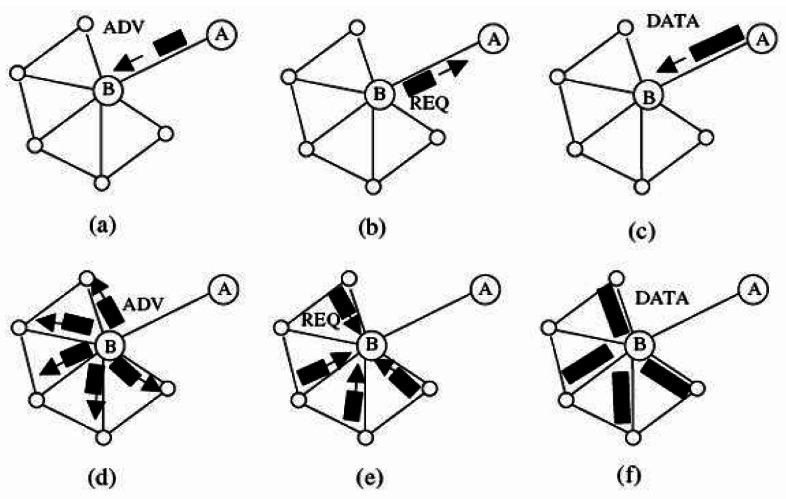
Process of data transmission in SPIN.

M-SPIN (Modified-SPIN) [44] is an improved version of SPIN (Sensor Protocols for Information via Negotiation) protocol [8] in which like in SPIN, data negotiation among the nodes is taken into consideration. It can distribute data between sensors by considering the limited energy resources of the nodes effectively. The nodes which run this protocol name their data by using some meta-data which are high level describers. Also, the nodes can decide about their communications with both the information of the application and their available resources. This can enable the sensors to distribute their data effectively. This protocol begins when a node achieves new data and decides to transmit it. Thus, it chooses a name for the new data and sends an advertisement message (ADV) to its neighbors. While receiving the ADV message, the neighbor node checks to see whether it has received or requested such data before or not. If not, the neighbor node transmits a request message (REQ) to the sender node and asks it to send its new data. Finally, the DATA message will be sent from the sender node and the process will be finished (Figure 1).

On the other hand, M-SPIN could reduce the number of packet transmissions in comparison with SPIN. SPIN performs negotiation before data transmission but in M-SPIN, the number of nodes which transmit REQ messages in response to ADV message is limited only to the nodes which are nearer to the sink. Also, the neighbors of the sink are identified by a distance discovery algorithm which operates based on the hop counts of the nodes to sink. Therefore, by finding the sink's neighbors, instead of broadcasting the data packet, it is transmitted toward the sink or its neighbors. The main problem of this approach is the considerable loads on sink's neighbors which may lead to their failure.

**Figure 2.**
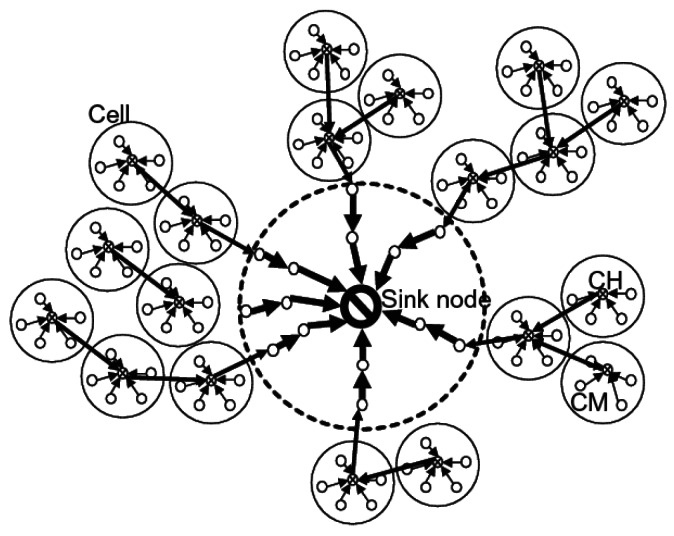
Hotspot area in hybrid routing scheme [54].

In [53] the authors proposed a hybrid routing algorithm which benefits from both hierarchical and flat routing mechanisms. Their proposed approach aims to solve the *hotspot* problem in WSNs. Hotspot in this approach is defined as the area in the interior of the maximum transmission distance of the sink node. In other word, the area of hotspot only contains the nodes which are located in the radio range of the sink. They have illustrated that failure of the nodes in the hotspot ceases the connection between sink and other nodes; hence, there should be an energy-efficient scheme to be aware about the energy level of the nodes in the hotspot area. The authors propose a flat routing approach in this area for selecting the most energy-efficient path and balancing energy among the nodes. Also, they have used a hierarchical structure for the nodes beyond the hotspot area. Figure 2 depicts this scheme. In the figure, the dotted circle indicates the hotspot area.

For selecting the next hop neighbor in their flat routing mechanism, they consider the links cost based on the remaining energy of the nodes. The calculation of link cost is performed as below:

$$\begin{array}{c} \text{linkcost}(\text{i},\text{j})=e_{s}(i)+e_{r}(j)\\ e_{s}(i)=\varepsilon_{1}{d_{i,j}}^{2}+\varepsilon_{2}\\ e_{r}(j)=\varepsilon_{3} \end{array}$$

In this equation, *linkcost* (i, j) is the energy cost of transmitting a unit of data from node i to node j. *e_s_*(*i*) is the consumed energy of node i for sending its data to node j which is proportional to the square of the distance between the sending node i and the receiving node j. *e_r_*(*j*) denotes the energy consumed by node j for receiving the data of node i. Finally, *ε*_1_, *ε*_2_ and *ε*_3_ are constant values which are characteristic of the sensor node's transmitting and receiving circuitry.

Beyond the hotspot area, the proposed scheme uses a clustering mechanism similar to LEACH [54] to reduce the transmission power by compressing the volume of data. The compression is done based on the correlation of data, since more correlation leads to more compression.

In addition to the mentioned approaches which focus on residual battery status, a number of works have prototyped the use of solar energy to power wireless sensor networks and balancing energy among the nodes [55–58]. In this area, although a lot of work has subsequently been put into the design and development of solar-powered sensor nodes, only a few makeshift topologies and routing protocols have been implemented.

The paper in [59] presents a scheme which uses solar energy efficiently. In this approach, each node operates in an energy-saving (ES) mode, if the node is short of residual energy; otherwise, the node operates in an energy-rich (ER) mode, and tries to construct an ER-backbone network consisting of fellow ER-nodes. Moreover, to choose the best next-hop node, they proposed ER-backbone-based geographic routing (ERB-GR) scheme, which is designed to balance energy and achieve low energy consumption.

In the ERB-GR scheme, all the ES-nodes try to route data in an energy-efficient manner, considering the remaining energy and the geographic location of their neighbors. Firstly, a node tries to route data directly to the ER-backbone by sending it to one of its ER-neighbors. However, if it has no ER-neighbors, it tries to find its most promising ES-neighbors. Additionally, this routing scheme is applied to some ER-nodes which have no ER-neighbors in the direction of the sink node. These ER-nodes should transmit data to one of their ES neighbors in an energy-efficient manner. In order to choose the best next-hop node in terms of energy-efficiency, ERB-GR calculates metrics called Progress and Cost Progress for each neighbor. From node *i's* point of view, Progress (i, j) and CostProgress (i, j) for a neighbor node *j* are calculated as follows:

$$\begin{array}{c} \text{Progress}(\text{i},\text{j})=D_{i}^{\text{sink}}-D_{j}^{\text{sink}}\\ \text{CostProgress}(\text{i},\text{j})=\frac{f(j)}{\text{Progress}(i,j)} \end{array}$$

Where $D_{i}^{\text{sink}}$ is the Euclidean distance from node *i* to the sink, $D_{j}^{\text{sink}}$ is the Euclidean distance from node *j* to the sink, and *f*(*j*) is proportional to the inverse of node *j*'s remaining energy, which expresses the degree of reluctance to forward a packet to node *j*. To calculate *f*(*j*), every node periodically broadcasts its energy level to its neighbors. Based on the values that it calculates, ERB-GR selects the next-hop node which has the smallest CostProgress (i, j) among all its neighbors.

#### 3.1.2. Energy Consumption Reduction

Clustering is known as an energy-efficient structure for data routing in WSNs. Hierarchal multi-hop routing algorithms successfully utilize data aggregation to decrease the volume of data flowing in the network. There are several data routing approaches in this area which proposed effective solutions for cluster formation and cluster head selection.

**Figure 3.**
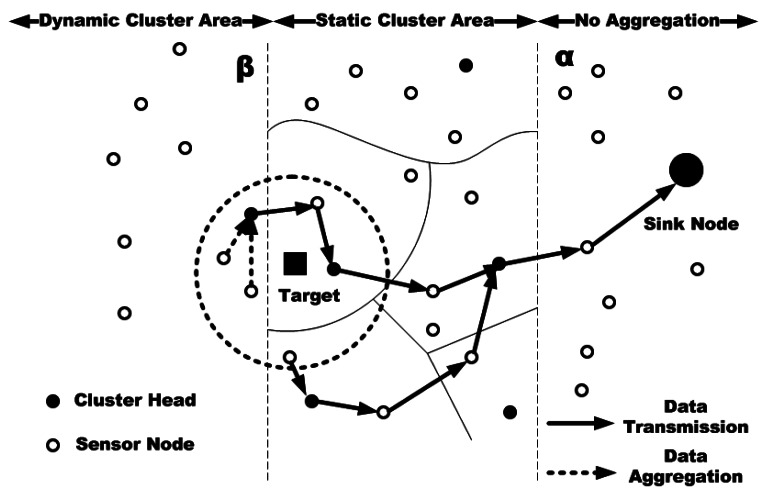
Combined Clustering-based data aggregation.

In [60], the proposed solution consists of two data aggregation mechanisms based on clustering: combined data aggregation and adaptive data aggregation. Combined data aggregation is able to use both static and dynamic clustering methods concurrently in the defined network according to the environmental variables of the network; therefore, it can make use of the advantages of both techniques simultaneously. In this scheme, the area of each clustering approach is not fixed and it may be changed according to the number of nodes in the network. In the initialization phase, first, a tree topology and a static cluster are constructed. Then, the nodes should select the data aggregation method they want to use. This selection is done based on two values of α and β. Value α illustrates the start of the static clustering based data aggregation and value β states the start of the dynamic clustering based data aggregation. Hence, the nodes whose values are between α and β send their data to the related cluster head using static clustering method. The values beyond the β will be aggregated using dynamic clustering method and the values below α will be directly sent to the sink without any aggregation. Therefore, by using this scheme, dynamic clustering is provided for the nodes far from the sink and the nodes near the sink which need low transmission power can send their data directly to the sink (Figure 3).

On the other hand, the adaptive data aggregation scheme can select a suitable clustering technique according to the state of network and application. In this scheme, each sensor node can adaptively change its data aggregation technique regarding the state of the network which can be affected by the number of targets. In this mechanism, the static cluster based aggregation is selected based on the network traffic. When the traffic is high, it chooses the static cluster based data aggregation and when it is low, the dynamic cluster based aggregation is selected. The decision for switching between the data aggregation techniques is made based on a threshold which is adjusted and decided at the BS.

The approach in [61] uses a three-tier architecture to propose a cluster-based routing algorithm. In this hierarchical scheme, the cluster formation is done before networking process. Cluster heads which are called gateways have more energy than other sensor nodes and it is assumed that they know the location of all other nodes. Thus, gateways nodes establish multi hop routes for data collection based on the maintained states of the nodes. In this approach, a TDMA like MAC is considered for data transmission between the sensor nodes and the gateway. A whole epoch is divided to some time slots and each node transmits it data in its own time slot. Also, all the nodes are notified by the gateway about the slot in which they should listen or transmit.

The sensor nodes in this approach can be in four states: sensing only, relaying only, sensing-relaying, and inactive. In the sensing only state, the nodes sense the environment and generate environmental data. In the relaying only state, the nodes go to listening phase and wait for receiving data of other nodes. In the sensing-relaying state, the nodes perform data generation while they are in listening phase, too. Finally, when a node carries out none of these functions, it is considered inactive.

Generated data of the sensor nodes are transmitted toward the gateway through the bidirectional links between the nodes. For performing an efficient data delivery, authors considered different costs for the paths; thus, the proposed routing approach selects the least cost path for data transmission from a node to the gateway. Furthermore, the gateway always monitors the energy level of the nodes and when it finds a node with insufficient energy to participate in routing operation, it immediately reroute the packet.

The aim of the authors in Hierarchical Geographic Multicast Routing (HGMR) for wireless sensor networks [62] is enhancing efficient data forwarding and increasing the scalability to a large-scale network. HGMR almost incorporates the key design concepts of the Geographic Multicast Routing (GMR) [46] and Hierarchical Rendezvous Point Multicast (HRPM) protocols [63] and optimizes these two routing protocols in the wireless sensor network environment.

HGMR, based on GMR, reduces the number of multicast packets from nodes to destination to conserve more energy. Moreover, it reduces the byte overhead of the packet which increases the average energy consumption in GMR; particularly, when the number of multicast members is high. For addressing this problem, it uses the idea of HRMP and divides the large groups of multicast members into multiple subgroups. On the other hand, HRMP is not efficient in packet transmission as it unicasts a same data packet to multiple sub-trees which consumes the energy of the nodes and overloads the bandwidth. Therefore, HRMP uses GMR's cost over progress optimizing broadcast approach in choosing the next relay nodes at each hop.

In the proposed approach, based on HRMP idea, the multicast group is divided into some subgroups. Then, the sensing area is again divided into several cells where in each cell there is an Access Point (AP) for managing the members of the cell. Also, there is a Rendezvous Point (RP) which is responsible to all Aps. Thus, when a node decides to transmit its data, it uses HRPM unicast-based forwarding mechanism to transmit its data to its AP along with the GMR idea in selecting the next hop node.

The GEAR (Geographic Energy Aware Routing) approach [1] replaces the network communications with limited geographical communications. The main idea of this routing approach is reducing the number of transmissions rather than broadcasting the packets to all the nodes. Also, data transmission toward the destination is performed by considering the cost of data transmission which is based on the energy level and distance to destination.

**Figure 4.**
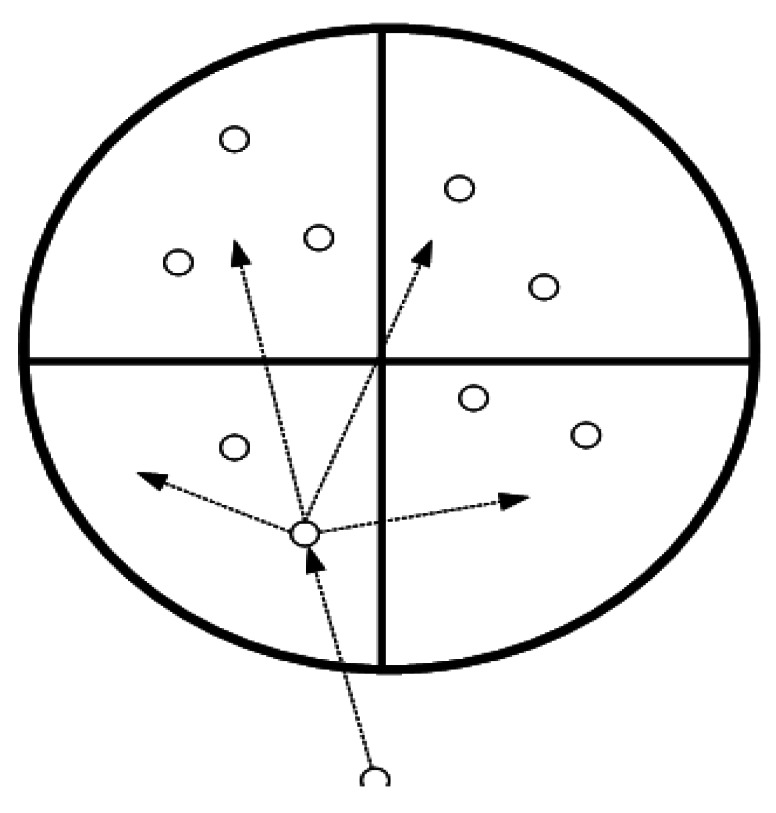
Recursive Geographic Forwarding Algorithm of GEAR (adapted from [1]).

In the proposed scheme, when users propagate their queries to the network, the queries which relates to a specific part of the network can be propagated to that area directly by GEAR algorithm. Thus, instead of broadcasting the message to the whole network, the queries are broadcasted only in their interested area. Therefore, this algorithm leads to an optimal propagation in applications using queries in geographic area level, Figure 4.

Data forwarding in this approach includes two levels:

1. Packet transmission to a specific area. In this level, each node upon receiving a packet, forwards the packet to the neighbor which is the nearest one to the destination. In some cases, where all its neighbors are further than itself (which is called hole), the neighbor with the best learning cost will be selected as the next hop. Learning cost is a proportion between remaining energy and distance to target which is considered in GEAR.
2. Second level is broadcasting the packets in the target area. Two modes of broadcasting are considered in this area. Restricted geographic flooding for scattered deployments and recursive flooding which acts by frequent splitting of the area for dense deployments.

The goal of authors in [20] when proposing the GAF (Geographical Adaptive Fidelity) approach is reducing the energy consumption by limiting the number of nodes in the routing process. In this approach, a virtual grid is considered in the covered area and each node is assigned a point in the grid structure by using a GPS receiver. GAF is a location-based approach but since the nodes are organized in several partitions (like clusters) and there are some leaders in each partition to transmit data to other nodes (like cluster heads), it can be considered as a hierarchical approach, too.

**Figure 5.**
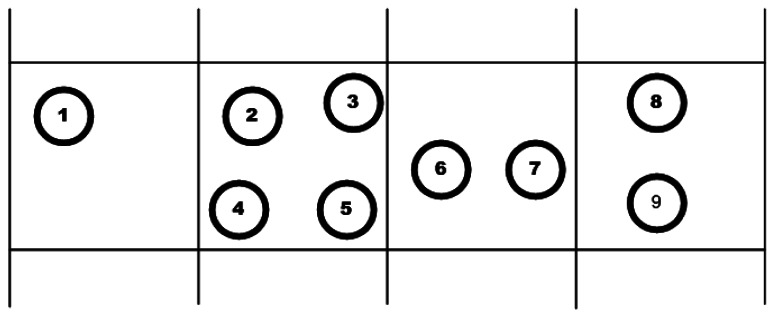
Example of virtual grid in GAF.

In the routing process of GAF, the cost of the nodes with the same points considered equal and they have assumed equivalent. Hence, several equivalent nodes can sleep during the routing process which can conserve considerable energy. Figure 5 depicts an example of this model. Any of nodes 2, 3, 4 and 5 can be the next hop for nodes 1. Thus, nodes 2, 3, 4 and 5 are equivalent and the non-selected nodes can sleep.

GAF defines tree states in its algorithm: *discovery, active, sleep*. In the *discover* state, the nodes determine their neighbors in the grid, the *active* state illustrates that the node is involved the routing process and the *sleep* state means that the radio of the nodes is turned off.

In this approach, the reliable mobility of the nodes is also supported. Each active node calculates the time in which it leaves the grid and sends a notification to its neighbors to inform them about the time. Thus, before the active node leaves, one of the sleeping nodes goes into active state to be replaced with the previous active node. For example, in Figure 5, if we consider node 2 as the active node which is going to leave its related area, before it leaves the area, any of nodes 3, 4 or 5 can replace it.

### 3.2. Delay-Aware Approaches

As illustrated before, according to the vital role of energy in sensor networks, any data routing approach in WSNs should be aware about the energy levels of the nodes beside its routing goal. Therefore, most of the proposed delay-aware data routing approaches considered energy in their mechanism, too.

Delay-aware approaches can be classified into two types: hard delay-aware and soft delay-aware. In hard delay-aware routing approaches, deterministic end-to-end delay bound should be supported and the main goal is ensuring the on-time delivery of real-time data, whereas in soft delay-aware routing, a probabilistic guarantee is required and the goal is just selecting the shortest available path.

#### 3.2.1. Hard Delay-Aware Approaches

The protocols in [14] and [64] are suitable for time-critical applications. In Threshold-Sensitive Energy Efficient Sensor Network Protocol (TEEN), the nodes sense the medium repeatedly, but data dissemination is done less frequently. A cluster head transmits a hard and a soft threshold to its cluster's members. A hard threshold enables the nodes to transmit their data only when it is in the required range of interest. Thus, this threshold can reduce a number of redundant and unnecessary messages in the network. On the other hand, soft threshold presents the changes in the sensed values. It allows the nodes to sleep in their idle times and wake up when the changes are greater than soft threshold. Therefore, sensor nodes can conserve considerable energy in their idle times. But the main problem of this approach is dependability of the algorithm on these thresholds. When there is no threshold, there is no communication between the nodes either. Furthermore, Adaptive Periodic TEEN (APTEEN) improved TEEN by using a TDMA like scheduling in which when a node does not sense data for a specific period of time; it will be obliged to sense and transmit data. In this approach, only the nodes which their sensed data are equal or greater than hard threshold can send their data. Moreover, such data is transmitted only when the value of that attribute differs by an amount at or beyond soft threshold. Furthermore, APTEEN considers a threshold for transmission, too. If the nodes do not transmit their data up to this threshold, they are forced to sense and retransmit the data. However, the main drawback of this approach is the complexity which is added to the basic function of the TEEN approach.

**Figure 6.**
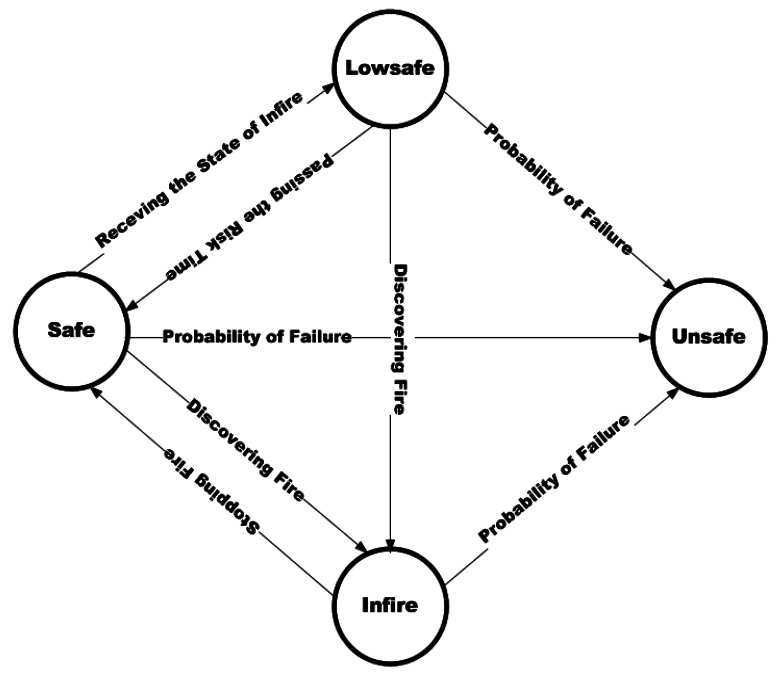
State diagram of the nodes in EAR.

Emergency-Adaptive Routing (EAR) which is proposed by authors in [48] is a real-time routing protocol in WSN for building fire emergencies and other similar applications. EAR computes the delay in its routing mechanism for selecting the next hop. Moreover, end-to-end delay from nodes to sink is calculated according to the delay of each hop. In this approach, each node can be in four specific states: *Safe* which means there is no fire, *Infire* when the nodes discover fire, *Lowsafe* states that the node is one hop away from the *Infire* node and finally *Unsafe* which illustrates the malfunctioning or failure of the node. Furthermore, there is a *State* which is used by the nodes to notify their current situation. When a node detects fire, it broadcasts the state of *Infire* to notify the occurrence of fire. The nodes which receive this message, mark themselves *Lowsafe* and propagate their status. Also, when a node finds that its residual energy is going to be finished, it broadcasts a message with the state of *Unsafe* to inform other nodes that it may be failed in the near future. Figure 6 presents the state transition diagram of each node in EAR.

In the initialization phase of EAR, it is assumed that sinks are not prone to failure. After deployment, an initialization message is broadcasted to the nodes containing the *height* parameter which demonstrates the hop count of the nodes to the sink. Each node upon receiving the packet, increments the *height* and forwards it to the next hop. In this process, the end-to-end delay which is considered the summation of the delays of each hop is calculated for path selection. *delay (sink, i)* as the delay of transmission from a node to sink is approximated as below:

$$\text{delay}(\text{sink},i)=\sum_{n=1}^{h}\text{Avg\_delay}=\sum_{n=1}^{h}(T_{c}+T_{t}+T_{q})*R$$

In the formula, *n* is the hop count from the sink to node *i, Tc* is the time taken in each hop to obtain the wireless channel with carrier sense delay and back-off delay. *Tt* is the time to transmit a packet which determined by channel bandwidth, packet length and the adopted coding scheme. *Tq* is the queuing delay which depends on the traffic load, and *R* is the retransmission count. When this process is finished, the shortest path with minimum delay from each node to sink is specified.

Energy-Efficient and Fast Data Gathering Protocols for Indoor Wireless Sensor Networks [12] focus on some specific applications which require prompt reactions. The proposed protocol considers the indoor environment and it suitable for alert services such as warning of poisonous gases in rooms. This paper, proposes two hierarchical protocols in the names of R-EERP and S-EERP based on LEACH with different clustering structures. In R-EERP nodes are deployed randomly, but in S-EERP their structure is sequential. In both protocols nodes are fixed during the cluster change time and their cluster head selection is done based on LEACH. In this approach, similar to TEEN, two threshold values named critical threshold and base threshold are defined. Base threshold demonstrates the minimum required value which should be sensed; thus, the values below this threshold are not acceptable. On the other hand, critical threshold relates to emergency situations and values above this threshold considers real-time values which cannot tolerate any delay. Therefore, cluster heads attempt to transmit such values with minimum delay.

In this approach, the sensed data of the nodes are limited and only the values which are greater than the base threshold are transmitted to the cluster heads. Also, when a cluster head receives a value beyond the critical threshold, it immediately and without waiting for other values transmits the data toward the base station to satisfy the emergency requirement of that data; otherwise, when it receives some data between base and critical thresholds, it performs required filtering and compression before transmitting data toward the sink.

In [65] the authors proposed a structure-free Real-time data Aggregation protocol (RAG) which makes use of two methods for temporal and spatial convergence of packets: Judiciously Waiting policy and Real-time Data-aware Anycasting policy. According to their simulation results, they have proved that RAG can be effective in term of end-to-end delay and energy-efficiency. In RAG, Judiciously Waiting policy is used to satisfy the on-time delivery of data packets. In this policy, the end-to-end delay which is the estimated time of data delivery is calculated by measuring estimated one hop delay including channel contentions and packet transmissions and queuing delay by using a time-stamping method. This scheme can effectively increase temporal convergence for data aggregation while performing delay-sensitive data delivery. The waiting time out (WT) for *R_h_* which is an intermediate node with *h* hop distance from the sink is calculated as below:

$$\text{WT}=\frac{\text{TTL}-\text{EED}}{1+(\frac{R_{h}-1}{R_{h}})}\times \alpha =\frac{\text{TTL}-(R_{h}\times \text{EHD})}{2-(\frac{1}{R_{h}})}\times \alpha$$

In the equation, the following factors are used for routing decision: TTL (Time-to-Live) which gives the remaining time of the packet to be received at the destination, EED (End-to-End Delay) which is the time required for delivering a packet from a node to sink and EHD (Estimated one-Hop Delay) including channel contentions, packet transmissions, and queuing delay.

On the other side, Real-time Data-aware Anycasting policy, by doing some computations, decides which next hop node achieves better aggregation performance while satisfying real-time requirements. In this policy, node *A* computes the required velocity based on the progress made toward the sink node and the packet's TTL before forwarding its data to the next hop. The computation is done as follows:

$$V_{\text{req}}=\frac{d(A,\text{Sink})}{\text{TTL}}$$

where *d* (*A, Sink*) is the Euclidean distance between node *A* and the sink node. Therefore, by satisfying the required velocity of each hop, the end-to-end deadline is addressed.

#### 3.2.2. Soft Delay-Aware Approaches

In [47] a simple least time and energy-efficient routing protocol named LEO is proposed which is able to select the shortest path and guaranty minimum time for routing. LEO uses absolute time rather than hope count to reduce both latency and congestion. For selecting the shortest path, first an initialization packet containing the absolute time of data delivery from node to sink and the remaining energy of the node is propagated to the network. Each node upon receiving the initialization packet, by using the information sent from the previous node, calculates the time required for a packet to reach the BS. This process is repeated until the entire network is covered. The outcome of the initialization phase is that, each node will know all its neighbors in its radio frequency (broadcast) region, time to reach the BS of each of neighbors and their residual energy. From such information, the node is able to determine the neighbor which has the least travel time to reach the BS and the highest remaining energy.

In this approach, the sensed data are classified as real-time or non-real-time. Real-time data are sent through the nodes with least travel time while non-real time data are sent through neighbor nodes with maximum available energy. Thus, the scheme is able to respond regarding the needed quality of service. The process of classifying and forwarding data at all the nodes repeats till the BS is reached. By doing so, it is assured that the data which has priority reaches the BS within the shortest time and regular type data is forwarded in such a way that the energy of the neighboring nodes reduces uniformly, thereby increasing the lifetime of the network. It is important to note that, if the majority of data are priority packets, then the network lifetime will be shorter. The reason is that, he same node will be selected for forwarding the data and it will die faster. To avoid such situations, a simple relation is proposed by authors as AX + BY, were A and B are constants, X, Y are node energy and time of data delivery values respectively. The values of A and B can be selectively decided as per the application and the type of data (priority or routine) is more in the network. This way, the problem of a node starving and losing energy quickly is overcome when the majority of the data are of priority type.

The approach in [66] proposed a fast data collection mobility-based mechanism in heterogeneous WSNs. The authors in this paper criticize the latency in previous mobility-based approaches caused by the delay in reaching the sensor nodes by the mobile sink. Thus, they have proposed a strategy for guiding the mobile sink in the sensing area to reduce the delay of data collection. In the proposed scheme, sensor nodes measure the local topology information while moving in the sensor fields and gradually transmit their positional information to the sink. Therefore, the sink(s) can be aware about the current positions of the nodes and adjusts its position according to the nodes' location and finds the shortest path for fast data delivery. For achieving this, two different schemes are proposed: *greedy scheme* in which the sink moves toward the regions with high densities and *aggregated scheme* for dealing with the aggregated regions in wider network areas. While the greedy scheme is appropriate for spatially balanced networks area, the aggregated scheme is useful for the areas which are geographically correlated.

In the proposed approach, while the nodes moving in the surrounding environment, they carry some local topology information about the sub-regions wherein they are mobile. Such information is measured by considering local density, corresponding position and time. Moreover, the significance of the information by using a ranking function is estimated. When the rank of the information goes down, it means that the information is not updated; therefore, the old information is updated with some new information. By using this mechanism, sensor nodes always transmit the updated information to the mobile sink which makes it aware about the current distribution of the nodes. Hence, it can reach and serve each sub-region quickly.

**Figure 7.**
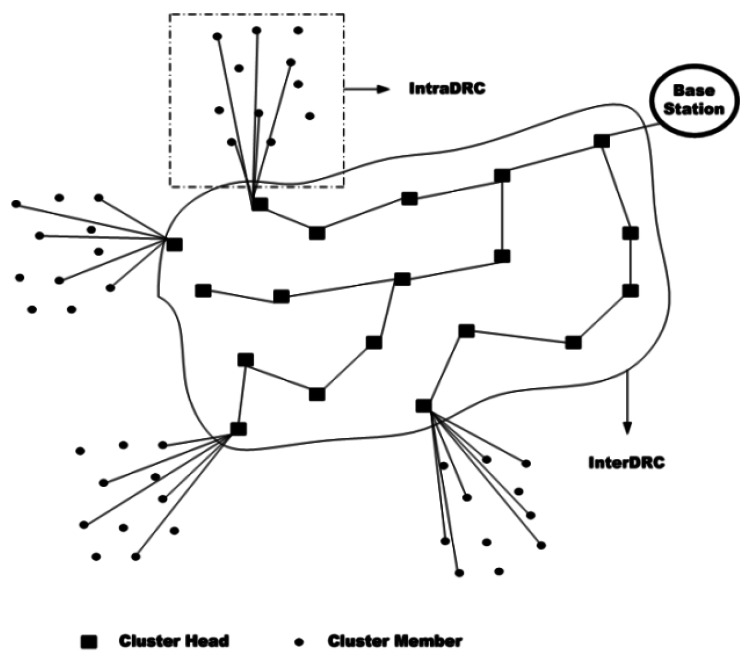
IntraDRC and InterDRC.

In [67] authors proposed a QoS-aware hierarchical data routing approach in WSNs. In this approach, the whole network is organized into multiple clusters wherein, each node upon sensing data, transmits its data to its cluster head. After receiving the data of the cluster members by the cluster heads and performing data aggregation, the results are sent to the base station through a routing tree. The whole approach consists of two main components: intra-cluster data reporting control (IntraDRC) and inter-cluster control (InterDRC) (Figure 7).

IntraDRC controls the nodes inside the clusters by selecting and scheduling data reporting nodes. Also, for data report scheduling, IntraDRC scheme uses CSMA/CA to support contention-based channel access mode and TDMA to support contention-free mode but the default mode of the scheme is based on the contention-based mode.

On the other hand, InterDRC establishes a data reporting tree between cluster heads to sink. For performing a data delivery from a node to sink, InterDRC considers two separate paths: one for energy-efficient delivery which is based on the traffic and another one for delay-sensitive reporting which selects the paths with minimum number of hops. The end-to-end delay in this approach is calculated as below:

$$D_{s=D^{\text{prop}}+(D^{\text{res}}+D^{\text{prop}})*h_{s}+D^{\text{agg}}*{ℋ}_{\text{int}}}$$

In this equation, *D^res^* is the residence time, *D^prop^* demonstrates the propagation time, *D^agg^* is the delay of data aggregation which is done in each cluster, *h_s_* presents the number of hops from nodes to sink and finally ℋ*_int_* denotes the number of intermediate clusters between the cluster heads and the sink in the data reporting tree.

The Data Gathering algorithm based on Mobile Agents (DGMA) [68] was proposed for cluster-based wireless sensor networks and it aims to reduce the network end-to-end delay and energy consumption. In DGMA, the whole network is considered as a cluster in which the base station is the cluster head. When a cluster head gathered the information of its cluster, it transmits the data to one of its neighbors which is the nearest node to the base station. This process is repeated until the results are received by the base station. Moreover, a mobile agent is considered in each cluster which passes over each hop for data collection. In this approach, cluster formation is done according to the occurrence of events. When there is no event, nodes can sleep but when an event occurs, the nodes which have detected the event are clustered and send their data to their cluster head. In this approach several states are considered for each node and the states of the nodes can be changed to another one according to some predefined thresholds. The defined threshold are: Basic Hard Threshold (BTH) to estimate the severity degree of the emergent events, Standard Hard Threshold (NTH) for waking the idle nodes up, Soft Threshold (ST) to mark the changes of data, Relative Exciting Threshold (RETT) to confirm the lifetime of the cluster heads and Absolute Exciting Threshold (AETT) to estimate the lifetime of the clusters. Also, the next hop selection in this algorithm is done based on the formula below:

$$\text{H}=\max\;\left(\alpha *\frac{E_{a}}{E_{\text{Max}}}+\beta *\frac{L_{\text{Max}}}{L_{a}}\right)*\zeta *I_{a}$$

In this equation, *E_a_* is the remaining energy of node a, *E_Max_* is the maximum energy of node *a, L_a_* denotes the path loss for node *a, I_a_* is the event intention and *α, β* and *ζ* are setting parameters.

In the low latency routing scheme of ERB-GR [59] (introduced in Section 3.1), if a node is operating in ER-mode and has at least one ER-neighbor, it tries to choose the best next-hop node, which is that with the smallest expected latency to the sink node, from among its ER neighbors. In this approach, each node controls its duty-cycle dynamically to keep the average power consumption rate lower than the average charging rate of solar power. Thus, each node has its own operating and sleeping schedule that depends on its duty-cycle. The other factor required to estimate the latency involved in delivering data along a path from a node *n_i_* to the sink via the neighbor node *n_j_*, is the expected number of hops in that path, since the expected waiting time will be encountered at every hop along the path. Finally, the expected latency from node *n_i_* to the sink node, when node *n_i_* sends data to neighbor node *n_j_*, $L_{\text{expected}}^{\text{total}}(j)$, can be calculated at node *n_i_* as follows:

$$L_{\text{expected}}^{\text{total}}\;(j)=L_{\text{expected}}^{\text{one–hop}}\;(j)\times H_{\text{expected}}^{\text{total}}\;(j)$$

where $L_{\text{expected}}^{\text{one–hop}}\;(j)$ and $H_{\text{expected}}^{\text{total}}\;(j)$ respectively are the expected latency per hop and the expected number of hops to the sink. Lastly, node *n_i_* chooses its ER-neighbor which has the smallest expected latency to the sink node, as the node to which to send data. This routing scheme calculates the expected latency of each ER-neighbor more accurately by using not only duty-cycle and geographic information about its one-hop neighbors but the same information about its two-hop neighbors. Since each one-hop neighbor also has information about its own one-hop neighbors, the node naturally can use the information about its two-hop neighbors.

### 3.3. Fault-Tolerant Approaches

In fault-tolerant data routing approaches, the main focus is providing sufficient reliability to satisfy the needed accuracy, but since using such mechanisms may reduce the energy of the nodes, most of the approaches in this category are aware about the energy level of nodes, too. In general, fault-tolerant approaches have two behaviors against faults: using some methods or adding different levels of redundancy to prevent faults or detecting and recovering the occurred faults.

#### 3.3.1. Fault Prevention

**Figure 8.**
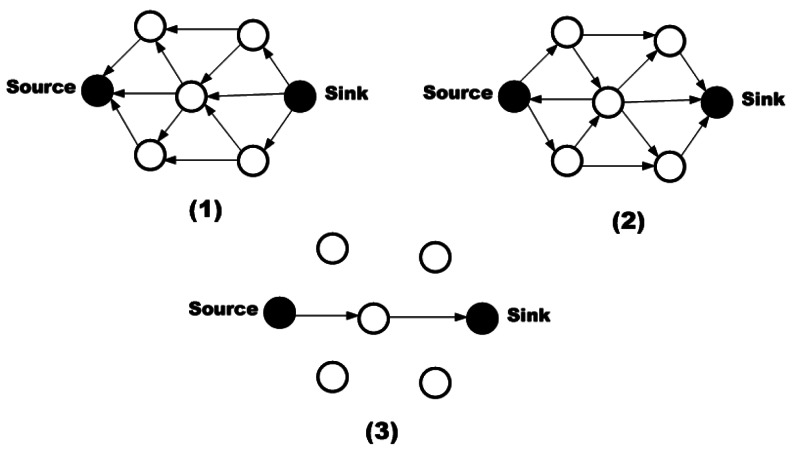
The operation of Directed Diffusion Algorithm.

In Directed Diffusion (DD) protocol [8] receivers and resources use some attributes for recognizing the produced or required information. The goal of this approach is finding an efficient multi-path route between senders and receivers to provide a strong tolerance against node failures. In this scheme, each task is represented as an interest and each interest is a set of attribute-value pairs. For performing a task, the related task will be propagated to the network. In DD, receiver nodes memorize the senders. Thus, a gradient which presents the direction of data flow and the status of request (which can be active, inactive or requiring update) is created. If a node can predicate the next hop from the previous gradients or geographical information, it sends the request only to the nodes which are able to reply to the request; otherwise, it should broadcast the request to all its neighboring nodes. When a node receives a request which is compatible with its data, it activates it sensors for collecting the required information and transmits the packet toward the requester (Figure 8).

In DD, data is stored in the intermediate nodes during the forwarding process toward the destination. In fact, this scheme is considered to prevent replication and circle generation. Moreover, the maintained information in the intermediates nodes can be used for in-network information processing. Moreover, when the sink node receives data from multiple paths, according to the quality of each path, it selects the best path and reinforces the source to send its data through this path. Furthermore, when a failure happens in the active path or the rate of data delivery is reduced, the sink selects another best path for data transmission.

Energy-efficient and Reliable routing Scheme (EARS) was also proposed in [69] to improve the directed diffusion protocol. This data routing approach selects the next hop neighbor by taking advantage of radio information. In EARS, instead of broadcasting the packets into the network, the most suitable neighbor among other candidates is selected and the data is transmitted only to that node. This strategy resulted in better energy consumption in EARS in comparison with Directed Diffusion. In this approach, each node uses the radio-aware metric of MAC layer to evaluate the quality of links. The lowest value of this metric demonstrates the lowest data rate and Frame Error Rate (FER) of MAC layer. Therefore, the neighbor with the lowest radio-aware metric will be the candidate of the next hop. MAC layer Radio-aware Metric (*C_lq_*) is computed as follows:

$$C_{lq}=\left[O+\frac{N_{b}}{r}\right]\frac{1}{1-E_{f}}$$

In this equation, *O* is channel access overhead, *N_b_* is number of bits in test frame, r denotes bit rate and *E_f_* is the frame error rate. Therefore, in EAR unlike DD which selects the next hop neighbors based on flooding the interest message, next hop nodes are selected according to their *C_lq_* which is maintained in the routing table. For efficient selection of the next hop neighbors and to perform more reliable data transmission, a Request to Send (RTS) packet is sent to the node with the minimum *C_lq_*. If the result of the request becomes positive, the node will be selected as the next hop; otherwise, another node with the least *C_lq_* among the remaining neighbors will be selected as the next hop. Also, it should be notified that, although EARS can be a reliable routing approach but in comparison with DD, it adds more delay to the routing process due to its dependency to the routing table.

In [70] the authors proposed a hierarchical structure wherein some relay nodes which have higher capability and power are selected as the cluster heads. Then, such relay nodes are placed in different regions of the sensing environment in a way that each of the sensor nodes is covered by one of these cluster heads. Moreover, for performing a fault tolerant and successful data delivery, the relay nodes are connected to each other in a separate network. In this approach, two heuristic mechanisms to specify the potential locations of the relay nodes are proposed: *grid based mechanism* and *intersection based mechanism*. In the grid based approach, the whole network is considered as a virtual grid and the relay nodes are placed at the center and corners of each cell. In large scale sensor networks where the number of deployed nodes in the sensing environment is high and finding the location of the sensor nodes is difficult, this mechanism seems to be useful. On the other hand, when the locations of the sensor nodes are known and they are deployed in specific places of sensing area, an intersection based mechanism can be used which can calculate the correct positions of the relay nodes wherein they can cover all the sensor nodes. According to the proposed formulation of the approach, among the potential relay nodes, some relay nodes which cannot be considered as a cluster head may be used, too. Such relay nodes are exploited to maintain the survivability of the relay nodes and improving the network lifetime. Moreover, the proposed routing scheme considers the energy level of the critical relay nodes to provide more reliable data transmission. The proposed approach is proper for the applications in which the sensor nodes are immobile and the routing schedule and the placement of the relay nodes are managed by the base station.

**Figure 9.**
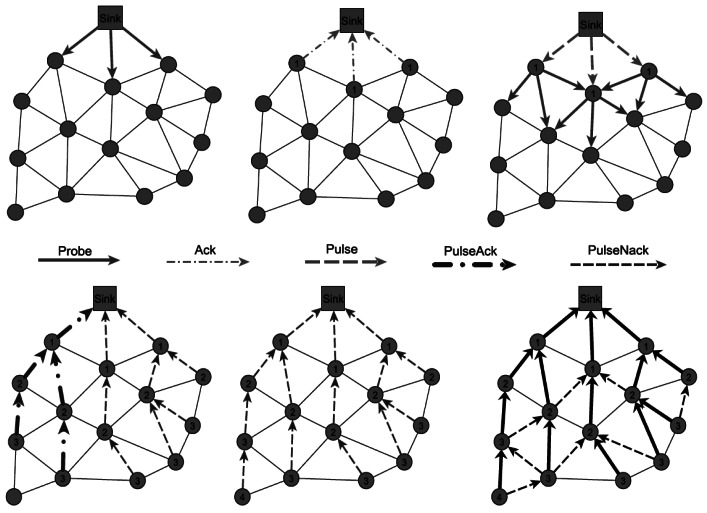
Process of shortest hop multi paths construction in SHM [71].

In [71] the authors proposed a distributed shortest hop multipath algorithm for satisfying fault-tolerance and load balancing in WSNs. The proposed Shortest Hop Multipath (SHM) algorithm is an improved version of Chang-Roberts distributed spanning tree (CRDST) algorithm which is able to construct a balanced spanning tree by defining and using different types of messages in a correct sequence order. CRDST uses two messages in its algorithm: *probe* message which is transmitted by the nodes for requesting a membership and *echo* message as an acknowledgement to the *probe* message. When all the nodes in the networks received their acknowledgement, the algorithm is finished. SHM criticizes this approach by notifying that the constructed spanning tree in CRDST is unbalanced and it cannot ensure Breadth-First-Search (BFS) tree formation after termination. Therefore, SHM ensures the BFS tree formation by utilization of β synchronizes approach [72]. The proposed algorithm is performed layer by layer and the operation of the nodes is switched to another layer when the nodes of one layer finished their functions (Figure 9).

**Figure 10.**
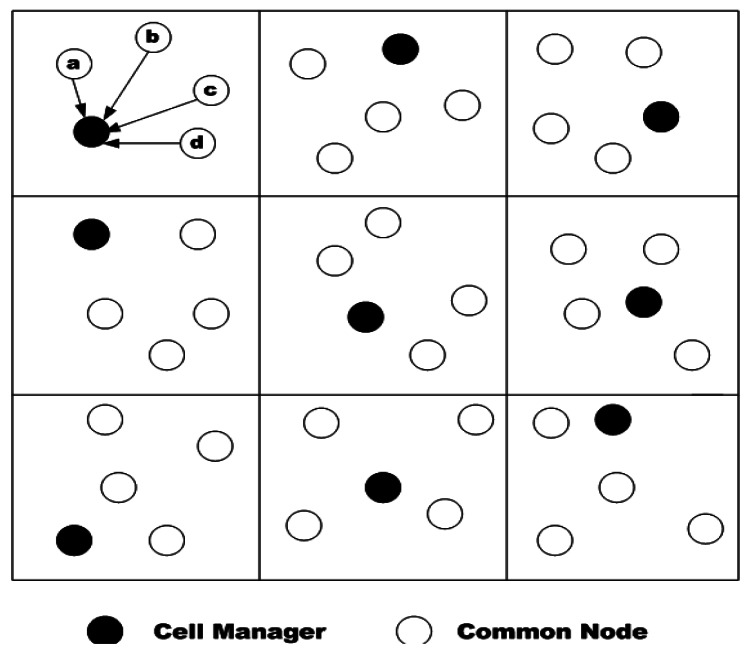
Virtual grid of cells.

SHM defines two types of nodes: initiator and non-initiator. The initiator node is the sink node which is used as the root of the spanning tree and other nodes (internal and leaf nodes) act as non-initiator nodes. Five types of messages are defined in this approach: *probe* message for parent request, *ack* message which is the acknowledgment of *probe, pulse* message which is sent by the sink node for specifying the proper level for sending the *probe* message and finally *pulseAck* and *pulseNack* as the acknowledgements of *pulse* message which have used for termination detection. Thus, the process of tree construction and shortest hop multi paths generation in SHM is done by taking advantage of these messages based on β synchronize approach. In this approach, initiator begins the next step when it receives all expected messages and the generation of BFS tree is done after each execution. Figure 10 depicts this process.

#### 3.3.2. Fault Recovery

An Adaptive routing protocol for fast Recovery from large-scale Failure (ARF) is proposed in [73] for applying a fast fault-tolerant mechanism in a routing tree for large scale sensor networks. ARF comprises a Routing Table, Table Manager, Link Estimator, Parent Selector, Cycle Detector, Forwarding Module, Routing Recoverer, Timer, and Dispatcher. In this approach, each node has its own parent, but it maintains a list of its neighbors as possible parents and counts its unsuccessful packet transmissions. Whenever a node cannot receive a signal from a neighbor, it considers the neighbor failed and extracts its ID from the list of possible parents. Also, when a parent of a node dies, the parent selector of the node starts the Routing Recoverer to select another node in the list of its possible parents as its parent. Moreover, when there isn't any alternative for replacing the parent, the node is considered orphan. In this case, the orphan node shortens its transmission interval and asks its neighbor nodes for a parent by a control message with the flag of orphan. When a neighbor node who has a reliable parent receives the orphan message, it attempts to help the nodes by shortening its transmission interval. Therefore, when the orphan receives a control message from a possible parent, it considers the sender as its parent and again extends its transmission interval.

The ARF algorithm depends on the parent node selection and routing tree construction. Whenever these two are carried out fast, the failure recovery is performed fast, too. The time complexity of initialization process of ARF is O (V) where V is the average number of neighbor nodes. Also, the process of link quality computing takes O (logV) and selecting back-up parent in parent procedure takes O (logV) time. Regarding the fact that a node can have maximum V−1 edges, the loop of finding parent runs V−1 times in a worst case. Therefore, the time complexity of parent procedure is O (V logV) in the worst case.

In [50] the authors proposed a cellular approach for fault detection and recovery. In the proposed mechanism, network area is separated into a virtual grid of cells. Each virtual grid is assigned a cell manager and a secondary manager as a back-up. The back-up managers handle the cell in case of cell manager's failure or when it cannot do its task (Figure 10). The cell managers in each cell perform and manage a fault-tolerant mechanism. At the initialization phase, selection of the cell managers is done based on their coordinates in the cell. In this mechanism, the node with the highest coordinates in the cell is selected as the cell manager and the node with the next highest coordinates is selected as the back-up manager. Later on, these selections are performed based on the residual energy of the nodes and the nodes with highest residual energy are selected as the managers. A cell manager receives the data of their members by one-hop communication and transmits it to its neighboring cell managers.

In this approach, fault recovery in done in each cell separately. When the energy level of a node goes below a threshold, the node is considered a failed node; therefore, it sends a message to the cell manager to inform it about its status; then, it changes its status to low computational mode. Moreover, when the energy level of the cell manager goes below the threshold, it sends a notification message to its members and the back-up manager. Therefore, the members consider the back-up manages as their new cell manager. On the other hand, a back-up manager considers itself the new cell manager and selects another node as the back-up manager upon receiving the notification message of the cell manager. For instance, in Figure 10, when the energy level of the cell manager goes below threshold, it sends a message to a, b, c and d to report its status. Hence, one of these nodes (like b) which have the highest residual energy will be selected as the cell manager. Next, among the remaining nodes, cell manager selects the one with highest residual energy as the back-up manager.

Distributed and Reliable Data Transmission (DRDT) scheme is proposed in [74] to enhance the reliability of data delivery. In DRDT, in case of packet loss, a neighbor node as a *helper node* which listens to packet transmission may handle the transmission of the lost packet. This is happened based on the link quality of the nodes. The quality of link (PRR) in this approach is calculated as below:

$$\text{PRR}(\text{d})={\left(1-\frac{1}{2}e^{-\frac{\gamma (d)}{2}\frac{1}{0.64}}\right)}^{16f-8l}$$

In this equation, d denotes the distance between two nodes, *γ*(*d*) demonstrates a Signal to Noise Ratio (SNR) for *d, f* presents the length of a frame, and *l* is the length of a preamble.

**Figure 11.**
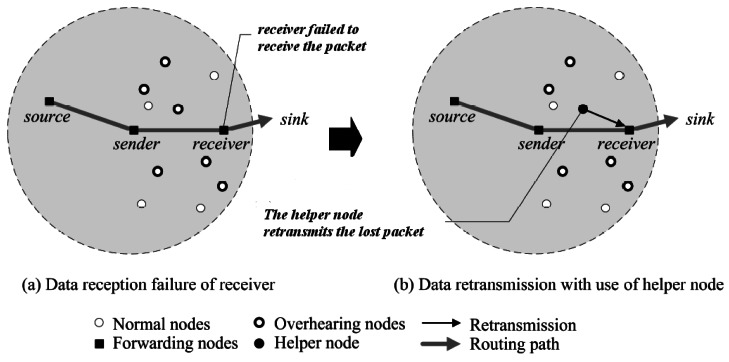
Retransmission process in DRDT [74].

In Figure 11, all the neighbor nodes which are in the radio range of the source and are able to listen to its transmission can be a helper node. Therefore, only when the data packet of the sender becomes lost and the PRR of the helper nodes is lower than the primary sender, it is asked to transmit the packet again. By using this strategy, the chance of packets to be received at the destination is increased and the number of attempts to perform packet retransmission is reduced.

For selecting a neighbor as a helper node, a node with the highest PRR to both sender and receiver is chosen. Therefore, the *helper value (H)* is defined which demonstrates the conditions of the neighbors. A neighbor with the highest *H* is the best candidate of the helper node. The helper value is calculated as below:

$$\begin{array}{c} \text{H}=w_{1}H_{\text{PRR}}(s,c)+w_{2}H_{\text{PRR}}(r,c)\\ w_{2}\geq w_{1}\geq 0\;\text{and}\;w_{1}+w_{2}=1 \end{array}$$

where *c* is the neighbor as a helper node and *w*_1_ and *w*_2_ denote the weighting values.

In this approach, in case of failure, each node which has listened to its neighbor transmission, checks the packet header to be sure that the destination is one of its neighbors. Then, it should wait to receive a possible command of retransmission. Thus, a Waiting Time (WT) is considered for a node which states how long it should wait for receiving the command of retransmission. It is computed as follows:

W=(1−H)×δ

where δ *is* the predefined maximum waiting time.

Dynamical jumping real-time fault-tolerant (DMRF) [75] is proposed for doing fast fault recovery. In this approach, when there is no fault, data routing is done in a normal hop by hop mode. But when a fault occurs, transmission is done in another mode named jumping mode. In this mode, the nodes use the transmission time of data packets along with the state of the next hop to compute the probability of packet reception at the next hop. This probability can be updated by getting some feedbacks from the downstream node. The goal of jumping mode is guarantying the packet delivery and reducing the delay of transmission in case of failure. To guarantee fault-tolerant transmission, M paths from source to destination which don't have any overlapping are considered in the initialization phase. Each node computes the distance and delay of message delivery for each path. Then, it selects k paths with minimum delay from the entire M paths and selects other M-K paths as alternative paths. The process of packet transmission in this approach is done by considering the deadline of the received packet, the transmission delay of the current path, the jumping probability and the congestion level.

In initialization phase, the nodes detect their neighbors and modify the list of neighbor nodes, Forwarding Candidate Set (FCS), table of jumping probability and the preliminary transmission route. On the other hand, in data forwarding phase, the proposed approach discovers failure and congestions. For detecting the failures, a packet is transmitted to FCS nodes and according to the received responses, the faulty nodes are specified. After this detection, the FCS nodes' information including their state, transmission rate and delay is updated.

In general, data forwarding in the proposed scheme is done in two modes: with failure or without failure. When there is no failure, the next hop node is selected from FCS based on the local information and the transmission rate of the packet. But in case of failure, it considers the remaining transmission time of packet to do jumping transmission. When the remaining time's value is below than jumping threshold, it switches to jumping mode. For selecting the next hop, the jumping probability is calculated. This calculation is done based on the results of previous jumping transmission or feedbacks from other nodes. By performing a jumping transmission, packets can pass over the failed nodes to be received by a proper node. Figure 12 depicts the process of jumping transmission.

## 11. Summary

In WSNs, data are routed from one node to another and to the base station by using different routing protocols. There are numerous data routing protocols designed for WSNs. According to the underlying network structure, data routing protocols in WSNs fall into three broad classes known as data-centric, hierarchical and location-based. In data-centric networks, each node has the same role and the duty of each node is transmitting the data packet toward the base station. In hierarchical networks, all the nodes are divided into several groups (clusters) and they have different responsibilities. The low level nodes as cluster members sense and collect data from the surrounding environment and the high level nodes as cluster heads manage and aggregate the data of their clusters. Location-based protocols using the positional information of the nodes to relay data to some desired regions. Such information can be achieved by some hardware devices such as GPS or by exchanging information between the nodes. On the other hand, most of the data routing protocols in WSNs are designed for a specific goal and aim to achieve one main goal. Also, some of them aim to achieve two or three goals together by considering a trade-off between the goals which can be considered as a new class of data routing as QoS-aware protocols. The main goals of data routing protocols in WSNs can be categorized as energy conservation, fast delivery and fault-tolerance. Each of these goals is addressed with different attitudes. Normally, energy efficiency can be improved by balancing the energy among the nodes or reducing the energy consumption of the nodes. Delay-awareness is provided with two attitudes: hard delay awareness in which on time data delivery has the highest priority or soft delay awareness which aims to route data through the shortest paths. Also, fault tolerant data routing is obtained in two forms: fault prevention to decrease the rate of fault occurrence and fault recovery to repair the failures.

Basically, these goals and attitudes are satisfied by proposing various methods or introducing a set of heuristics into the routing algorithms; for example, energy conservation in energy-efficient data routing approaches is achieved by considering the residual energy of the nodes during routing mechanism, selecting the least energy consuming paths through considering some metrics such as minimum number of hops, controlling the transmission ratio, reducing number of packet transmission or by managing the duty cycle carefully; Fast delivery is provided typically by delay-based scheduling of node transmission queues and selecting the routes with minimum delay; Fault tolerance is addressed in the reviewed algorithms by maintaining multiple routing paths and using an available one on-demand, retransmissions or switching the next hops in case of failures.

Moreover, each of the data routing protocols in WSNs has some operational characteristics in their routing mechanism which are used to achieve the desired goal. Here, we briefly introduce some of the main operational factors which are effective on data routing mechanisms:

### Mobility

In some applications, sensor nodes or the sink(s) can be mobile. When sensor nodes are mobile, they can configure their position to contribute in balancing energy consumption in some regions that contains high traffic load and make the network partitioning easier [76,77]. Also, when the sink is mobile [51,66], it can approach the nodes and gather the information of the nodes for balancing energy consumption among the nodes. In this condition, data can be sent periodically or it can be delayed until the sink changes its position to be in a shorter distance with the node; hence, shortening the transmission distance reduces the energy consumption. Of course, it should be noted that deploying mobile sink and sensor nodes increase the deployment of WSN.

### Number of sinks

According to the limited bandwidth of the sink, it may become the bottleneck of the data monitoring and leads to traffic congestion in case of simultaneous packet transmissions to a unique sink. By using multiple sinks, the nodes can have multiple targets to send their data to (Figure 12). Therefore, according to the required routing goal (such as energy or delay), the most appropriate sink can be selected or for performing fault-tolerant data routing, other sinks can be used as alternatives in case of failures [1,51,64].

### Data aggregation

**Figure 13.**
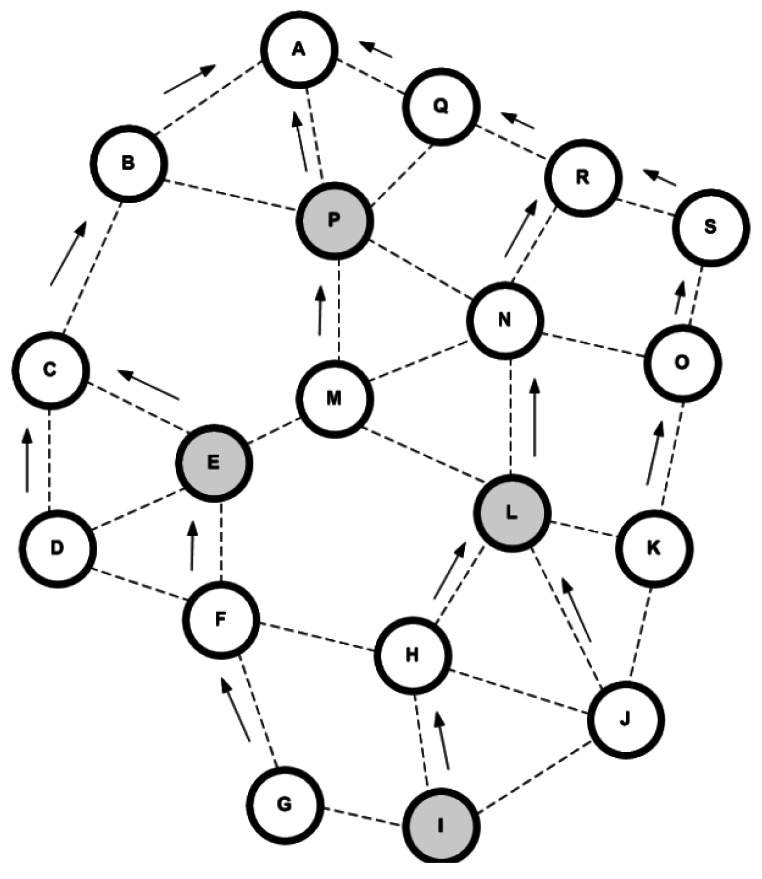
Data aggregation in WSNs.

In sensor networks, since sensor nodes might generate significant redundant data, similar packets from multiple nodes can be aggregated so that the number of transmissions would be reduced. When a node receives the results of other nodes, it combines the results, removes the redundant data, aggregates the data into one single packet and sends this packet toward the sink node. This process is continued until the final result is received by the sink node. Data aggregation is one of the most significant techniques which can be used to achieve energy efficiency and traffic optimization in routing operation [47,52,60,65]. For example, in Figure 13, all the nodes with gray colors can aggregate their received data packets.

Table 1 illustrates the main goals of some well known and current data routing approaches in WSNs according to their features. Of course, some approaches may have other goals in addition to their main goal, but in this table, only the main goal of the approaches is specified.

## 12. Conclusions

In this paper, data routing goals of the existing approaches are categorized into three main types: energy conservation, fast delivery and fault-tolerance. Moreover, the most famous and recent data routing approaches based on their attitudes and features are reviewed and compared in each goal category. According to this overview, most of the proposed data routing algorithms consider the energy of the nodes as an unavoidable factor in proposing a data routing approach in WSNs (HGMR, M-SPIN). Some of them consider delays (TEEN, EAR) and a number of them aim to provide fault tolerance (ARF, DMRF). However, by increasing the application of WSNs, the functions of sensor nodes will be so highlighted and they may be requested to do more complicated task. Therefore, scalable solutions which can perform data routing by considering multi-objective QoS requirements is greatly required for WSNs.

## Figures and Tables

**Figure 12.**
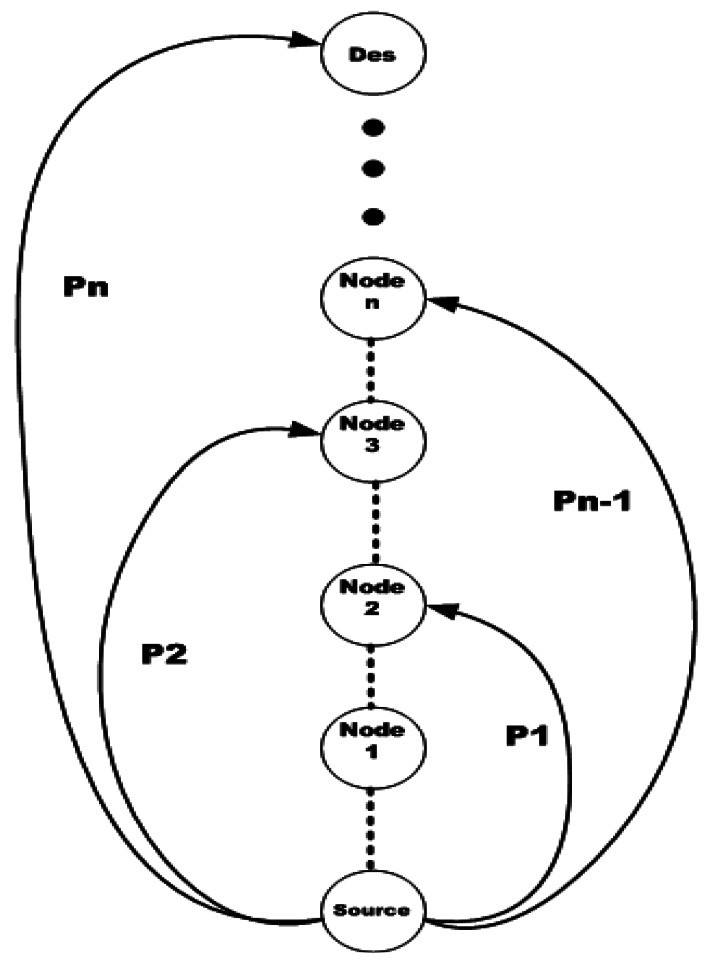
Jumping Transmission in DMRF.

**Figure 12.**
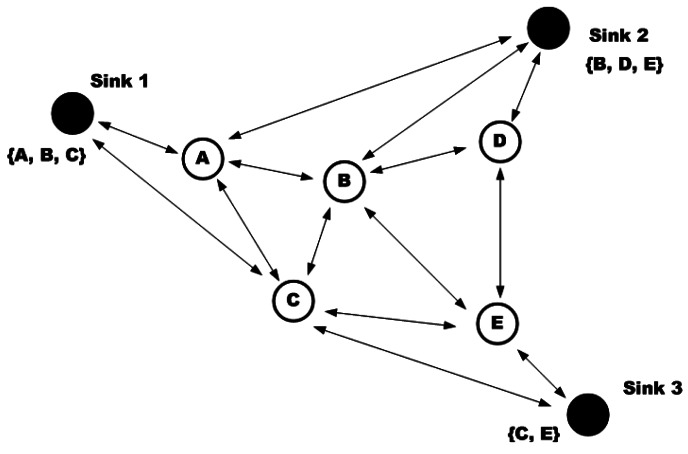
Using multiple sinks for data routing.

**Table 1.** Features and goals of data routing approaches.

**Protocol**	**Classification**	**NO of Sinks**	**Mobility**	**Data Aggregation**	**Energy Conservation**	**Fast Delivery**	**Fault-Tolerance**
M-SPIN [44]	Data-centric	1	Possible	Yes	√		
DD [10]	Data-centric	1 or more	Limited	Yes			√
Hybrid Clustering [60]	Hierarchical	1	Possible	Yes	√		
EAR [48]	Data-centric	1 or more	No	No		√	
SHM [71]	Data-centric	1	No	Yes			√
LEO [47]	Data-centric	1	No	Yes		√	
HGMR [62]	Location-based	1	No	No	√		
TEEN [14]/APTEEN [64]	Hierarchical	1	No	Yes		√	
DMRF [75]	Data-centric	1	No	No			√
GEAR [1]	Location-based	1 or more	Limited	No	√		
DGMA [68]	Hierarchical	1	Yes	No		√	
RAG [65]	Data-centric	1	No	Yes		√	
GAF [20]	Location-based	1	Limited	No	√		
ARF [73]	Data-centric	1	No	No			√
EAGR [19]	Location-based	1	Limited	No	√		
Fault tolerant Design [70]	Hierarchical	1	No	Yes			√
QoS-aware [67]	Hierarchical	1	No	Yes		√	
GESC [52]	Hierarchical	1	No	Yes	√		
Energy-efficient and Fast [12]	Hierarchical	1	No	Yes		√	
EARS [69]	Data-centric	1 or more	Limited	Yes			√
